# Exploring the role of neutrophil extracellular traps in neuroblastoma: identification of molecular subtypes and prognostic implications

**DOI:** 10.3389/fonc.2024.1361871

**Published:** 2024-11-07

**Authors:** Can Qi, Ziwei Zhao, Lin Chen, Le Wang, Yun Zhou, Guochen Duan

**Affiliations:** ^1^ Study Office of Pediatric and Thoracic Surgery, Hebei Medical University, Shijiazhuang, Hebei, China; ^2^ Department of Pediatric Surgery, Children's Hospital of Hebei Province, Shijiazhuang, China

**Keywords:** neuroblastoma, neutrophil extracellular traps, inflammation, prognosis, immune

## Abstract

**Background:**

Cancer cells induce neutrophil extracellular traps (NETs) to promote tumor progression and metastasis. However, only a few studies have focused on the role of NETs in Neuroblastoma (NB).

**Methods:**

First, based on the expression of NET-related genes, consensus clustering analysis was conducted to cluster NB samples into different subtypes. Differential analysis was performed to identify DEGs between subtypes. Functional items and related pathways of DEGs were identified using enrichment analysis. Univariate Cox analysis and the LASSO algorithm were used to identify biomarkers for prognosis. Furthermore, independent prognostic analysis was performed. Immune infiltration analysis was performed to identify differential immune cells. Finally, the verification of prognostic model genes were taken by the immunohistochemical staining and quantitative real-time PCR.

**Results:**

Consensus clustering analysis demonstrated that NB samples were clustered into two subtypes. There were 125 DEGs between the two subtypes of NB. Moreover, the enrichment analysis results showed that the DEGs were mainly associated with ‘external side of plasma membrane,’ ‘immune receptor activity’ ‘regulation of leukocyte migration’ GO items. There were also several GO items related to neutrophils, such as regulation of neutrophil migration and differentiation. KEGG pathways revealed that the DEGs were correlated with in immunity-related activities, including ‘Complement and coagulation cascades,’ ‘Neutrophil extracellular trap formation, ‘T cell receptor signaling pathway,’ ‘PD-L1 expression and PD-1 checkpoint pathway in cancer’ and so on. A total of five biomarkers,[Selenoprotein P1 (SEPP1), Fibrinogen-like protein 2 (FGL2), NK cell lectin-like receptor K1 (KLRK1), ATP-binding cassette transporters 6(ABCA6) and Galectins(GAL)], were screened, and a risk model based on the biomarkers was created. Furthermore, a nomogram for forecasting the survival rates of patients with NB was established based on the risk score, age at diagnosis, and MYCN status. Eight differential immune cells (CD8 + T cells, resting mast cells, etc.) were acquired between the two risk subgroups. The expression levels of five prognostic model genes at the protein and mRNA were verified and all results were consistent with the results of our bioinformatics analysis.

**Conclusion:**

We initially found that five NET-related genes were significantly differentially expressed in NETs-associated molecular isoforms and two Netrg molecular isoforms were found to be associated with poorer prognosis. This stratification might provide insight into the prediction of prognosis and ideal immunotherapy strategies for patients with NB. However, we also noted that the formation of NETs is a complex biological process involving the regulation of multiple cytokines and cellular interactions. Therefore, the exact roles of these genes and their specific mechanisms in the formation of NETs and the development of NB still need to be further investigated.

## 1 Introduction

Neuroblastoma (NB), an embryonic tumor originating from neural crest stem cells, is the most common extracranial malignancy in children and accounts for 15% of cancer-related deaths (1, 2). Despite advances in therapies such as immunotherapy, stem cell rescue, and transplantation, many high-risk NB patients still have poor prognosis (3, 4). Therefore, reliable biomarkers are urgently required to improve the prognostic evaluation and therapeutic effects of NB.

It is well known that the tumor microenvironment (TME) and immune components can interfere with tumor progression and metastasis (5–7). Neutrophils are the most abundant inflammatory cells in the TME and play an irreplaceable role in the response of tumor cells (8). Neutrophils within tumors are often termed tumor-associated neutrophils (TANs), which release neutrophil extracellular traps (NETs) into the TME (9, 10). NETs, a web-like structure, were first discovered by Volker Brinkmann and Arturo Zychlinsky in 2004 (10). They are released by neutrophils and can kill bacteria, protozoa, and viruses. It is composed of nuclear or mitochondrial DNA fibers, granular antimicrobial enzymes, and histones. NETosis is the process by which neutrophils extrude NETs. NETosis is a new type of cell death characterized by the release of decondensed chromatin and granular contents into the extracellular (10). NETs have been reported to play vital roles in infectious conditions, host defense mechanisms, thrombosis, wound healing, coagulation disorders, atherosclerosis and so on (10). The function of NETs in tumors was first proposed in pediatric Ewing sarcoma specimens in 2013 (11). The study is the first to show that TANs in Ewing sarcoma were activated to release NETs and to explore the possible role of NETs in cancer. Increasing evidence has demonstrated that NETs play key roles in TME, tumor cell awakening, migration, and invasion capacity (12, 13). Recently, several studies have revealed that NETs are involved in cancer progression and metastasis in multiple malignancies such as lung adenocarcinoma and breast cancer and so on (14–17). However, the prognostic value of and relationship between NET-related genes (NETRGs) and NB have not been fully clarified.

In this study, we initially searched for novel prognostic biomarkers or therapeutic targets of NETRGs to improve prognosis and guide therapy using bioinformatic analysis methods based on the NB public database.

## 2 Materials and methods

### 2.1 Data sources

The GSE85047 dataset for NB was obtained from the GEO online database and contained 276 NB samples with clinical and survival information (18). Moreover, an external validation set, including 150 NB samples with corresponding survival information, was acquired from the TARGET online database (https://ocg.cancer.gov/programs/target/) to verify the risk model. Furthermore, 136 NETRGs were obtained from the published literature (19).

### 2.2 Subtypes of NB samples identification

Based on the expression of NETRGs, a consensus clustering analysis was performed on all NB samples in the GSE85047 dataset, and the best clustering parameter was selected using the ConsensusClusterPlus (v 1.58.0) package (20). Furthermore, PCA was used to evaluate the distribution of the subtypes.

### 2.3 The differential expression analysis and enrichment analysis

In our study, DEGs between clusters of NB samples were acquired using the limma (v 3.54.0) package (21) (p.adj < 0.05, |log_2_FC| > 1). A heat map and volcano map of DEGs were plotted using the pheatmap (v 1.0.12) and ggplot2 (v 3.3.5) (22) packages, respectively. To further understand the related biological functions and signaling pathways of DEGs, GO and KEGG enrichment analyses were conducted using the clusterProfiler (v 4.2.2) package (23) (p.adj < 0.05).

### 2.4 Identification and verification of biomarkers

Subsequently, based on the above DEGs, univariate Cox analysis was conducted to screen for candidate genes related to NB prognosis (HR ≠ 1 and p < 0.01). Furthermore, the LASSO algorithm was used to identify biomarkers. Based on the expression of the above biomarkers, a risk model was created, and the samples in the GSE85047 and external validation set (TARGET) were classified into high- and low-risk groups, respectively, using the optimum cut-off value of the risk score ( $\text{risk score = }\sum_{1}^{\text{n}}{\text{coef(gene}}_{\text{i}})\;*\;{\text{expression(gene}}_{\text{i}})$ ). In addition, K-M survival curves and ROC curves (1-, 2-, and 3-year) were plotted. In addition, the differences in the risk scores between the different clinical indicator subgroups, including the International Neuroblastoma Staging System (INSS) stage, amplification status of MYCN oncogene (MYCN status), age at diagnosis, and progression, were further analyzed using the Wilcoxon or Kruskal-Wallis test methods.

### 2.5 Independent prognostic analysis

Furthermore, clinical features (INSS, MYCN status, etc.) and risk scores were included in univariate Cox analysis. In addition, multivariate Cox analysis was performed on the clinical features acquired by univariate Cox analysis to determine independent prognostic factors (p < 0.05). Subsequently, a nomogram for predicting the survival rates of patients with NB (1, 2- and 3-year) was created. Calibration and ROC curves were used to verify the validity of the nomograms.

### 2.6 The immune microenvironment analysis

A method for using gene expression signatures to infer stromal and immune cell ratios in 269 tumour samples (24). Using the R package estimate algorithm, it is possible to estimate the stromal score, immune score and ESTIMATE score of a tumour sample based on the expression data, used to represent the presence of stromal and immune cells. Moreover, differences in the three scores between the two subgroups were compared. The relationships between these three scores and the risk scores were analyzed. To further evaluate the infiltration of immune cells in NB samples, the CIBERSORT algorithm was used (25). CIBERSORT is a universal computational method for quantifying cellular components from bulk tissue gene expression profiles (GEPs). CIBERSORT combines support vector regression with *a priori* knowledge of purified leukocyte subpopulation expression profiles to accurately estimate the immunocomposition of tumour biopsies (26). We first performed a comprehensive analysis of immune cells in the 269 tumour samples collected. Based on the screening criteria that immune cells were not detected in more than 75% of the samples, we excluded some cell types. Ultimately, a total of 13 samples with TANs and their associated gene expression were included in the subsequent analyses. and the differential immune cells between the two subgroups were compared using the Wilcoxon test (p < 0.05). The relationship between the risk score and differential immune cells was computed using Spearman analysis.

### 2.7 Immunohistochemical staining

Paraffin specimens of neuroblastoma were collected from the Pathology Department of Hebei Children’s Hospital, which included 25 Non high-risk neuroblastoma samples and 7 high-risk neuroblastoma samples. The inclusion criteria were as follows: ①The patients were pathologically diagnosed with neuroblastoma. ②Patients were aged 0–18 years. ③Complete clinical data could be collected. ④Patients were without anti-tumor therapy, such as radiotherapy and chemotherapy. Exclusion criteria: ①clinical data could not be collected completely. ②Patients with complicated diseases and other tumors before surgery were excluded. ③Patients received the treatment, such as radiation or chemotherapy. These samples were from the tissue bank of pathology department, which ensures the integrity and representativeness of the samples. All samples selected were rigorously reviewed by pathologists and confirmed as neuroblastoma. In the process of sample collection and use, we strictly abide by the relevant ethical codes and laws and regulations, obtain the approval of the hospital ethics committee, and ensure the protection of patient privacy. All the staining steps were performed with the standard protocol. The sections were incubated with the SEPP1(1:100, PA5-50786, Thermofisher, USA),FGL2(1:50, PA554306, Thermofisher, USA),KLRK1(1:200, 14-5878-82, Thermofisher, USA),ABCA6(1:500, PA5-96236, Thermofisher, USA),GAL(1:500, PA5-62069, Thermofisher, USA)antibody. The stained sections were scored by two independent pathologists. The random 5 fields of view from each section were selected by Image J software to determine the average optical density value and statistical analysis was taken between the high-risk and Non high-risk neuroblastoma samples by SPSS 19.0 (IBM Inc.)

### 2.8 Quantitative real-time PCR

Two pair of freshly frozen High risk and Non-high risk tissues from patients with neuroblastoma were obtained after surgical resections between June 2023 and December 2023 in Children’s Hospital of Hebei Provinces. Total RNA was extracted from the tissue samples using TRIzol (Invitrogen). The RNA(1 µg) of each sample was used for reverse transcription with PrimeScript RT reagent (Takara). Then PCR was performed using Power SYBR^®^ Green PCR Master Mix (TaKaRa), according to the manufacturer’s instructions. The primer sequences for quantitative PCR were SEPP1: Forward:5’-CGTTGGAAGTGGTTGTGAC-3’; Reverse5’-CCATTGGAGTTTAGCATTGG -3’;FGL2: Forward:5’-AAATGTTCAAAGTGTCCCAGCCAAG-3’; Reverse5’-TGCCTATTGCGTAGTAGTCAGAGC -3’;KLRK1: Forward:5’- GGTATGAGAGCCAGGCTTCTTG -3’; Reverse 5’- GAATGGAGCCATCTTCCCACTG -3’;ABCA6: Forward:5’-CGCCATCGCAAGATTAGTGAGTG-3’; Reverse5’-CATCCAGGAGCAAGACAGGTGAG -3’;GAL: Forward:5’- GCTCGCCTCCCTCCTCCTC -3’; Reverse 5’- TCTTGTCGCTGAATGACCTGTGG -3’. Relative expression was normalized and 2^-ΔΔCt^ method was carried out to calculate the relative mRNA level. Experiments data were performed at least three times and the difference between two groups was compared by the Student’s t test. p<0.05 was considered to indicate statistical significance.

## 3 Results

### 3.1 Two subtypes of NB were obtained

**Figure 1:**
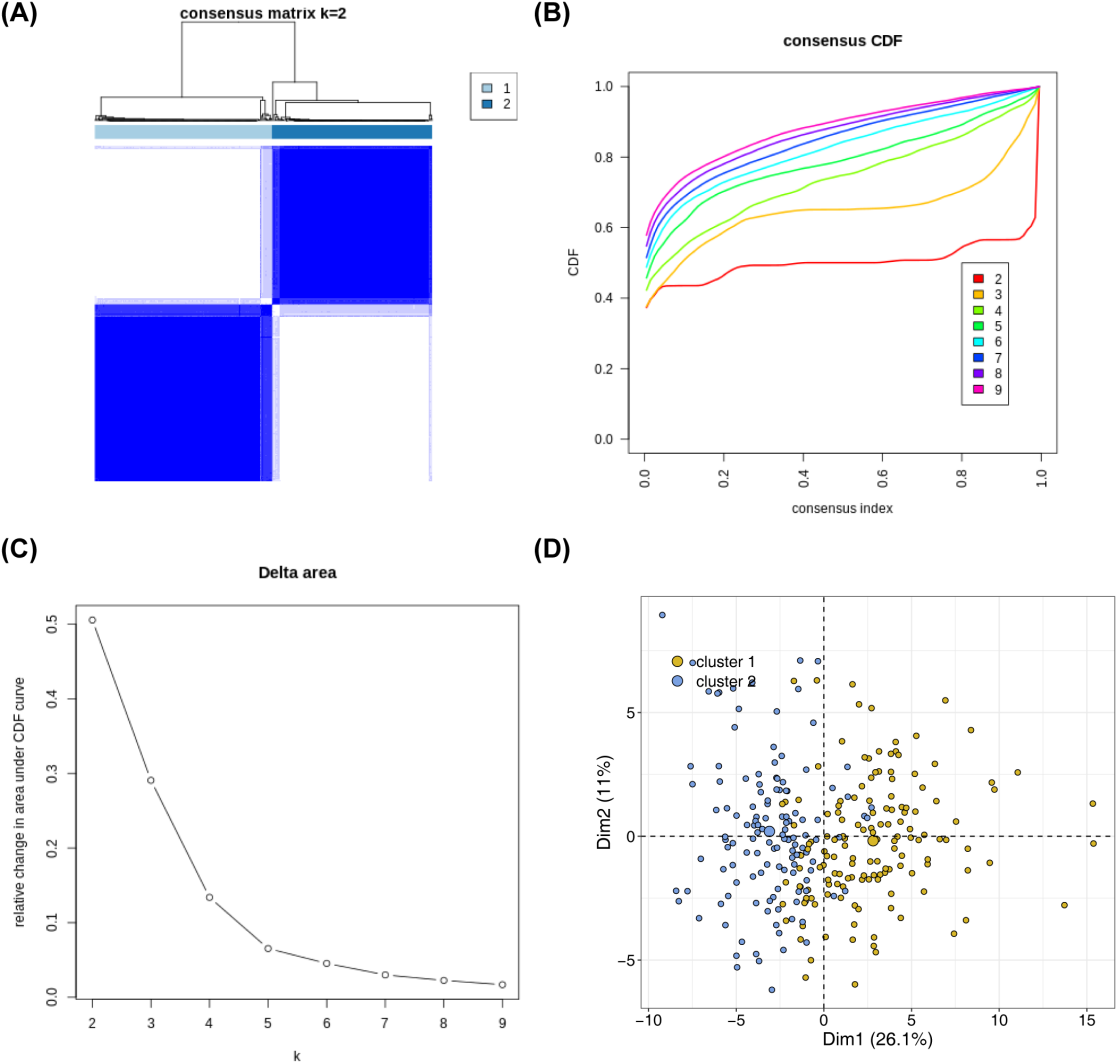
Identification of NETs -related subtypes **(A)** Consensus matrix heat map based on the expression of 136 NETs-related genes using R packege ConsensusClusterPlus on the NBL samples in the training set. **(B)** Cumulative distribution of CDF to determine the optimal number of subtypes **(C)** Delta area plot showing the relative change of areas under the CDF curve **(D)** Principal component analysis graphs generated based on cluster 1 and cluster 2 in the training set after classification into 2 subtypes.

Based on the expression of 136 NETs-related genes, the NBL samples in the training set were consistently clustered using R packege ConsensusClusterPlus, and subtypes were determined by the cumulative distribution function (CDF). The consensus clustering analysis results showed that clustering into two subtypes (K = 2) was the most suitable: Cluster 1 had 145 samples, and Cluster 2 had 131 samples (**Figures 1A–C**). The PCA results demonstrated obvious differences in the distribution of the subtypes (**Figure 1D**).

### 3.2 Differential gene expression and functional enrichment in NB subtypes highlight immune-related pathways and neutrophil activities

**Figure 2:**
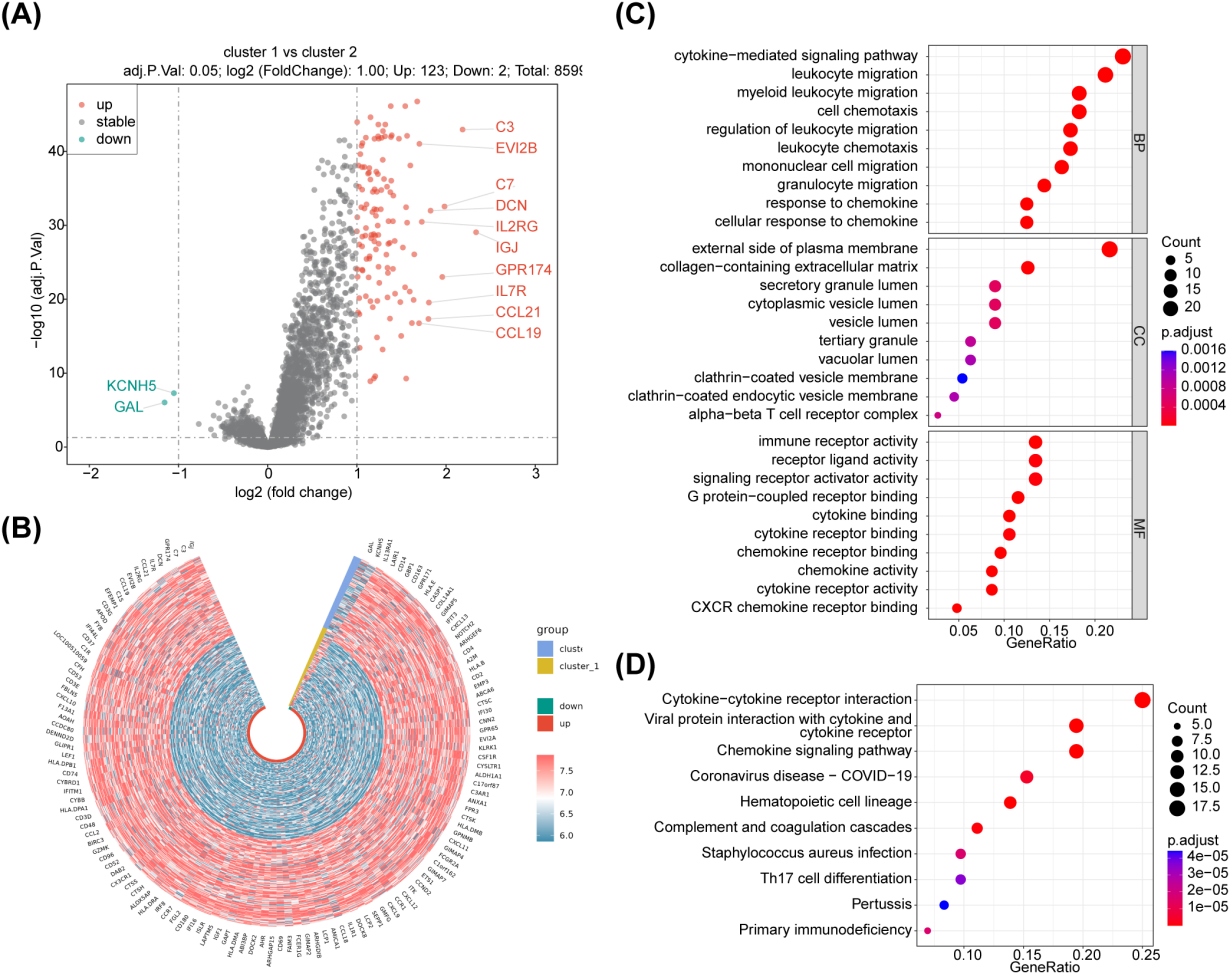
Identification of differentially expressed genes associated with NETs, were plotted using R package ggpubr and pheatmap, enrichment analysis of DEG, respectively. **(A)** The volcano maps, there were 125 differentially expressed genes between cluster 1 vs cluster 2 groups, of which 123 were up-regulated expressed genes and 2 were down-regulated expressed genes. **(B)** The heatmaps,the red dots on the graph indicate up-regulated genes, the blue dots indicate down-regulated genes, the black ones are non-significant, and the more skewed towards the upper left and right corners of the graph, the greater the multiplicity of differences in significance. **(C)** GO and **(D)** KEGG enrichment analyses,125 NETs-related differential genes for GO functional annotation.

Analysis of variance (cluster 1 (N=145) vs cluster 2 (N=131)) was performed on the training set data using the R package limma with differential gene screening criteria of p.adj<0.05 & |log2FC|>1. The results showed that 125 DEGs were found between the two subtypes of NB samples (**Figure 2A**; **Supplementary Table 1**). The expression heatmap of the DEGs is shown in **Figure 2B**. Moreover, functional enrichment analyses were performed to further investigate the functions performed by NETs-related differential genes. Enrichment analyses based on KEGG and GO databases were performed using the R package clusterProfiler package and the human gene annotation package org.Hs.eg.db to search for functions and related pathways common to a large number of genes within the gene set. The enrichment analysis results showed that the DEGs were mainly associated with ‘external side of plasma membrane,’ ‘myeloid leukocyte migration,’ ‘immune receptor activity’ ‘regulation of leukocyte migration’ GO items. There were also several GO items related to neutrophils, such as regulation of neutrophil migration and differentiation. KEGG pathways revealed that the DEGs were correlated with in immunity-related activities, including ‘Complement and coagulation cascades,’ ‘Primary immunodeficiency’ ‘Neutrophil extracellular trap formation, ‘T cell receptor signaling pathway,’ ‘PD-L1 expression and PD-1 checkpoint pathway in cancer’ and so on. (**Figures 2C, D**; **Supplementary Tables 2**, **3**).

### 3.3 Five prognostic biomarkers and risk model for neuroblastoma survival accurately predict clinical outcomes

**Figure 3:**
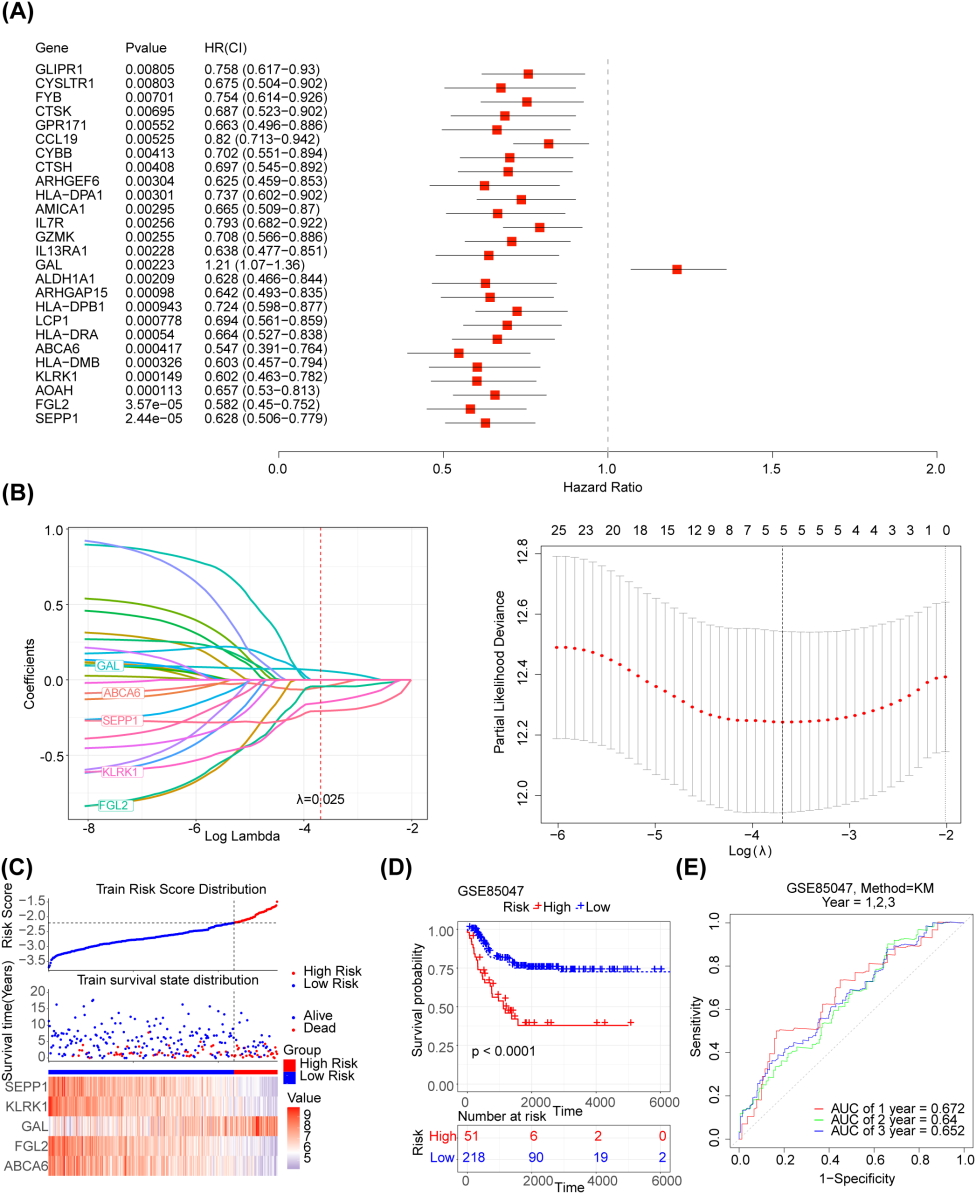
Development of the NETs -related prognostic signature in the training set **(A)** Forest plot of 26 NETs-related genes correlated with OS. **(B1-2)** The LASSO Cox regression analysis was performed depending on the optimal λ value. **(C)** Distribution of prognostic index in different risk groups, survival status of patients in different risk groups and the heatmap of prognostic gene. **(D)** The Kaplan-Meier curves showed that significant differences were identified for OS between these two risk groups. **(E)** ROC curves evaluating the sensitivity and specificity of the NETs-based prognostic model.

**Figure 4:**
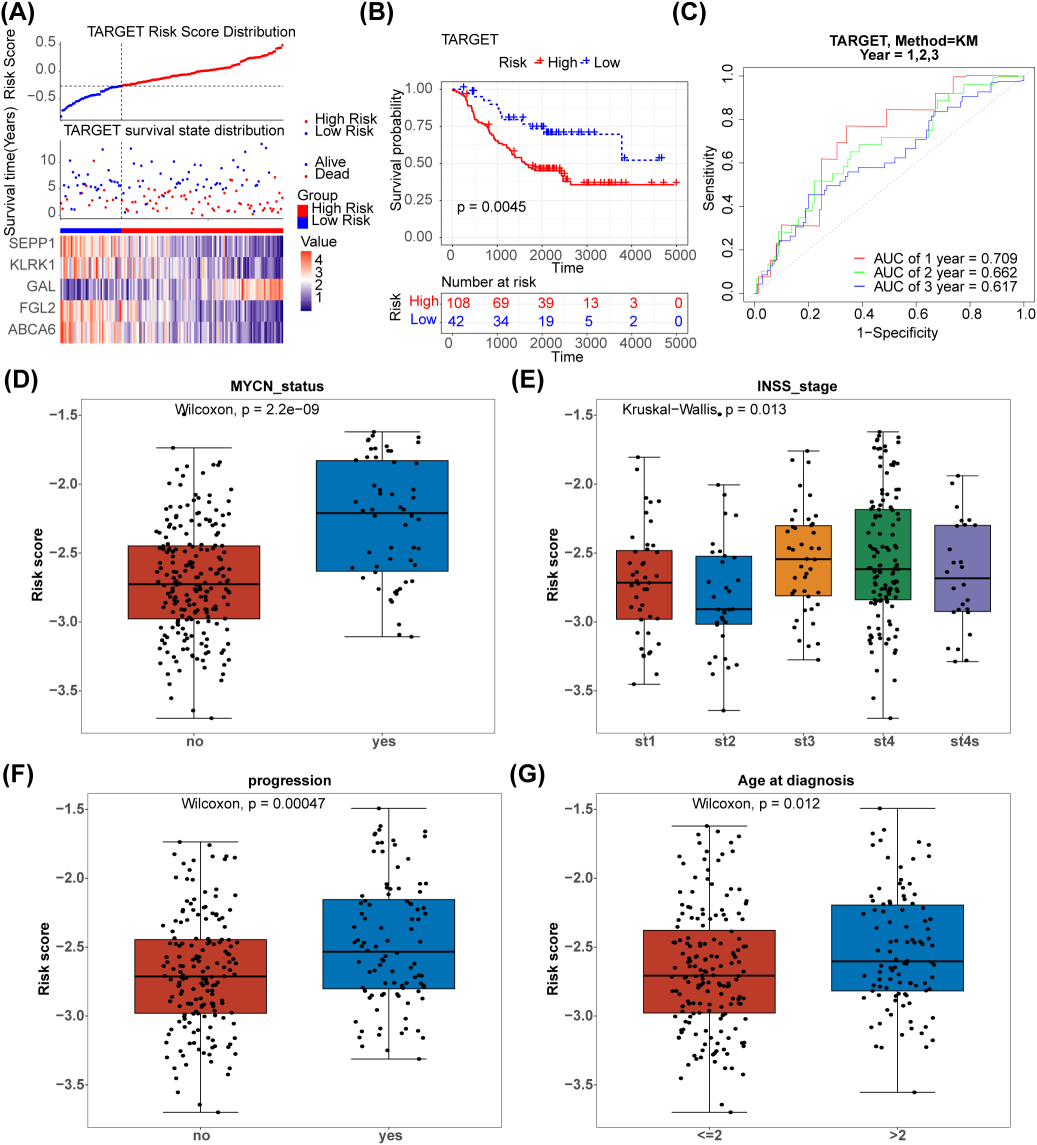
Validation of the NETs -based signature in the testing set.**(A)** Distribution of TARGET prognostic index in high risk group (n = 108) and low risk group (n = 42), survival status of patients in high risk group (n = 108) and low risk group (n = 42) and the heatmap of prognostic gene. **(B)** The Kaplan-Meier curves showed that significant differences were identified for OS between high risk group (n = 108) and low risk group (n = 42). **(C)** ROC curves evaluating the sensitivity and specificity of the NETs-based prognostic model(TARGET). **(D–G)** The association between clinical traits and risk score of NB.

The 125 NETs-related differential genes described above were used to construct the NB prognosis-related risk score. The data used were those from patients who had both gene expression information from tumour samples and complete survival information, and a final total of 269 patients were included in the subsequent analyses. First, the relationship between candidate gene expression and patients’ overall survival (OS) was analysed using a one-way Cox regression model. A total of 26 prognostic candidate genes were identified using the univariate Cox analysis (**Figure 3A**). Five biomarkers, [Selenoprotein P1 (SEPP1), Fibrinogen-like protein 2 (FGL2), NK cell lectin-like receptor K1 (KLRK1), ATP-binding cassette transporters 6(ABCA6) and Galectins(GAL)],were predicted using the LASSO algorithm (**Figure 3B**). The linear combination of the five genes and their coefficients in the model was used as the risk score. In the GSE85047 dataset, NB samples were classified into high- (51 samples) and low-risk (218 samples) groups, with an increase in the risk score and number of dead patients. After a risk score assessment based on the scores,we found that the expression of SEPP1, FGL2, KLRK1, and ABCA6 was upregulated, and that of GAL was downregulated in the low-risk group (**Figure 3C**). In addition, there was a distinct difference in survival between these two subgroups (p < 0.05), and the survival rate of the low-risk group was higher (**Figure 3D**). The AUC values (1-, 2-, and 3-year) were all > 0.6, demonstrating that the risk score could better predict the survival status of patients with NB (**Figure 3E**). Moreover, we verified the risk model in the external validation set (TARGET) and found that the results were consistent with those of the GSE85047 dataset (**Figures 4A–C**). There were distinct differences in the risk scores between the different groups for the four clinical features, including MYCN status (YES and NO), age at diagnosis (>2 and ≤ 2), INSS stage (st1, st2, st3, st4, and st4s), and progression (YES and NO) (**Figures 4D–G**). In addition, the Kaplan-Meier survival curve analysis data were conducted to explore the relationship between the expression level of each gene and survival in neuroblastoma patients(**Supplementary Figure S1**). According to the expression level of risk model genes, the samples were divided into the high and low expression groups. There were survival differences between the high and low expression groups of SEPP1, FGL2, KLRK1, ABCA6 and GAL. The survival probability of SEPP1, FGL2, KLRK1 and ABCA6 in the low expression group was significantly lower than that in the high expression group, and these genes were protective factors for NB, while GAL showed the opposite trend and was a risk factor for NB, which could be correlated with the results of single factor analysis. So each gene of the models was correlated with NB prognosis.

### 3.4 Development and validation of a nomogram for accurate survival prediction in neuroblastoma patients

**Figure 5:**
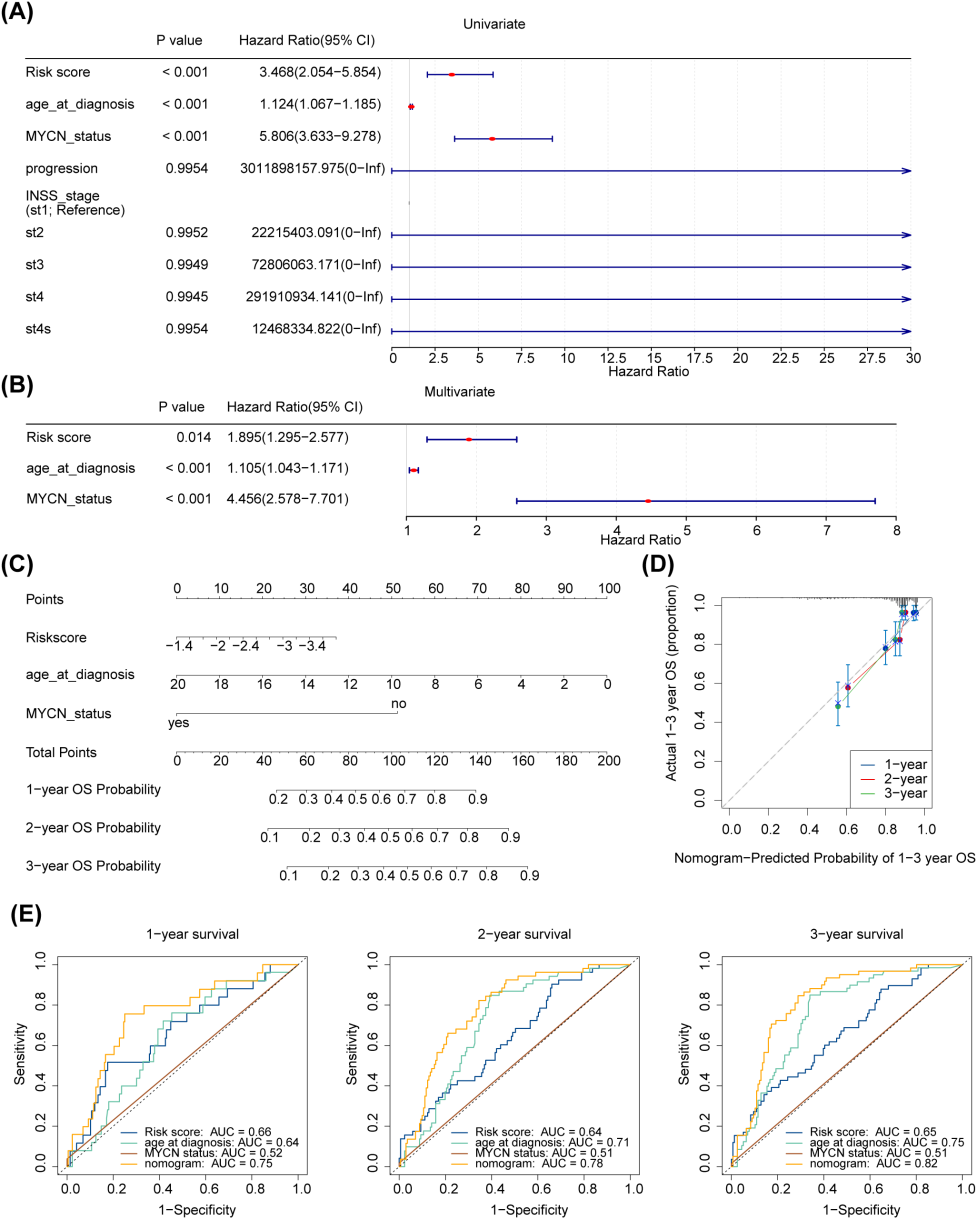
Independent prognostic analysis of risk scores and clinical parameters. **(A, B)** Forest plot of univariable and multivariate Cox model for survival in NB. “Age_at_diagnosis”: age at diagnosis; “MYCN_status”: the status of the MYCN gene in the tumour cells of a patient with neuroblastoma, a proto-oncogene associated with tumour growth and spread that is amplified in some neuroblastomas and is associated with aggressiveness and poor prognosis of the disease. “PROGRESSION”: in medicine and biology, the development or worsening of a disease or condition. “INSS_stage”: the abbreviation for international neuroblastoma staging system (INSS). INSS stages are as follows (St: Stage), Stage 1: The tumour is confined to the primary site, can be completely resected, and the patient is 18 months of age or older or the tumour diameter is less than or equal to 5 cm; Stage 2A: The tumour is partially resected and the ipsilateral residual tumour does not cross the midline; Stage 2B: partial resection of the tumour with ipsilateral residual tumour crossing the midline; Stage 3: Incomplete resection of the tumour due to invasion of major vascular structures or tumour crossing the midline with residual tumour; Stage 4: Tumour has distant metastases to other parts of the body; Stage 4S: Low risk of metastasis under certain conditions, usually seen in infants. **(C)** A nomogram to predict 1-year, 2-years, and 3-years survival rates in NB patients. **(D)** Nomogram-Predicted Probability of 1-3 year OS. **(E1–3)** Survival-dependent receiver operating characteristic curves for risk score, nomogram, and clinical pathological characteristics.

Further analysing the relationship between age at diagnosis, mycn proto-oncogene amplification status, disease stage according to the International Neuroblastoma Staging System (INSS), cancer progression and risk scores. The risk score, age at diagnosis, and MYCN status were screened using univariate Cox analysis (p < 0.05) (**Figure 5A**). Furthermore, age at diagnosis, risk score, and MYCN status were found to be independent prognostic factors (**Figure 5B**). A nomogram for survival forecasting in patients with NB (1, 2- and 3-year) was created based on these independent prognostic factors (**Figure 5C**). The calibration and ROC curves indicated that the nomogram had favorable predictive ability (**Figures 5D, E**).

### 3.5 Immunological landscape and cell composition delineate risk stratification in neuroblastoma

**Figure 6:**
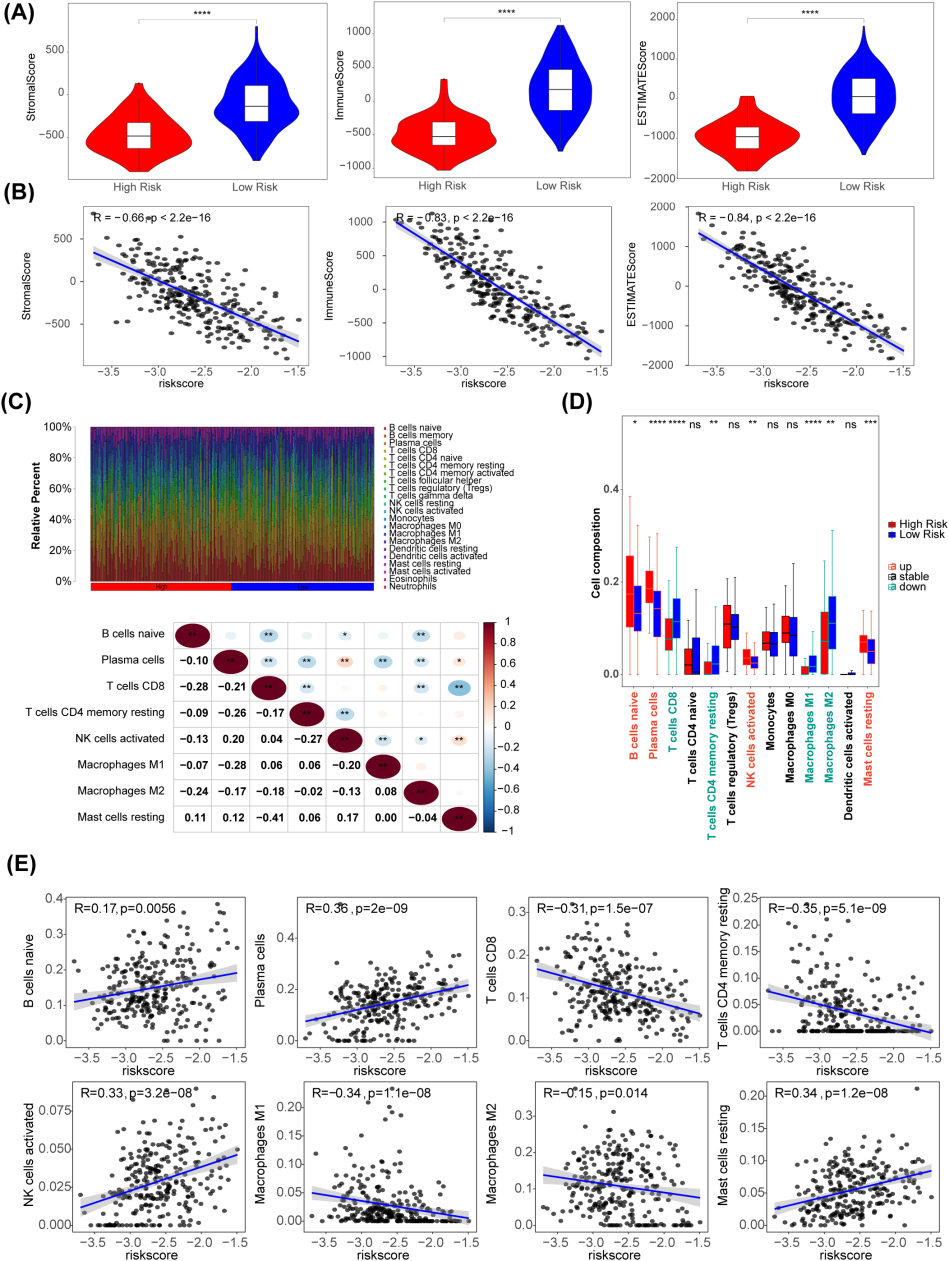
Immune-related characteristics in the low- and high-risk score groups. **(A)** Violin plots of stromal score, IMMUNE score, and ESTIMATE score for the training set samples in the high (n=51) and low (n=218) risk groups based on the training set using R package estimate. **(B)** The correlation between risk scores and stomal score, immune score, ESTIMATE score. **(C)** The distribution proportion of immune cell abundance in 269 sample. **(D)** Differences in immune cell infiltration between the low (n=218) and high (n=51) risk score groups. **(E)** The correlations of eight immune cells and risk scores. *p<0.05, ** p<0.01, *** p<0.001, **** p<0.0001, ns p>0.05.

Using the R package estimate algorithm, it is possible to estimate the stromal score, immune score and ESTIMATE score of a tumour sample based on the expression data, used to represent the presence of stromal and immune cells. The results are as follows, the ESTIMATE, immune, and stromal scores were significantly higher in the low-risk group (P < 0.05) (**Figure 6A**). Meanwhile, there were significant negative associations between these three scores and risk score (|R| > 0.65) (**Figure 6B**). The distribution of immune cell abundance in each sample is shown in **Figure 6C**. Furthermore, eight different immune cells (naive B cells, plasma cells, CD8 T cells, resting memory CD4 T cells, activated NK cells, M1 Macrophages, M2 Macrophages, and resting mast cells) were acquired between the two risk subgroups (**Figure 6D**). The cell abundance of neutrophils analyzed by CIBERSORT algorithm was 0 in more than 75% of the samples. Neutrophil was filtered out and did not display with boxplot. In addition, plasma cells had the strongest positive association with the risk score (R = 0.36), and there was a strong negative relationship between the risk score and CD4 memory resting T cells (R = -0.35) (**Figure 6E**).

### 3.6 Experimental validation of prognostic model genes in neuroblastoma confirms bioinformatic predictions

**Figure 7:**
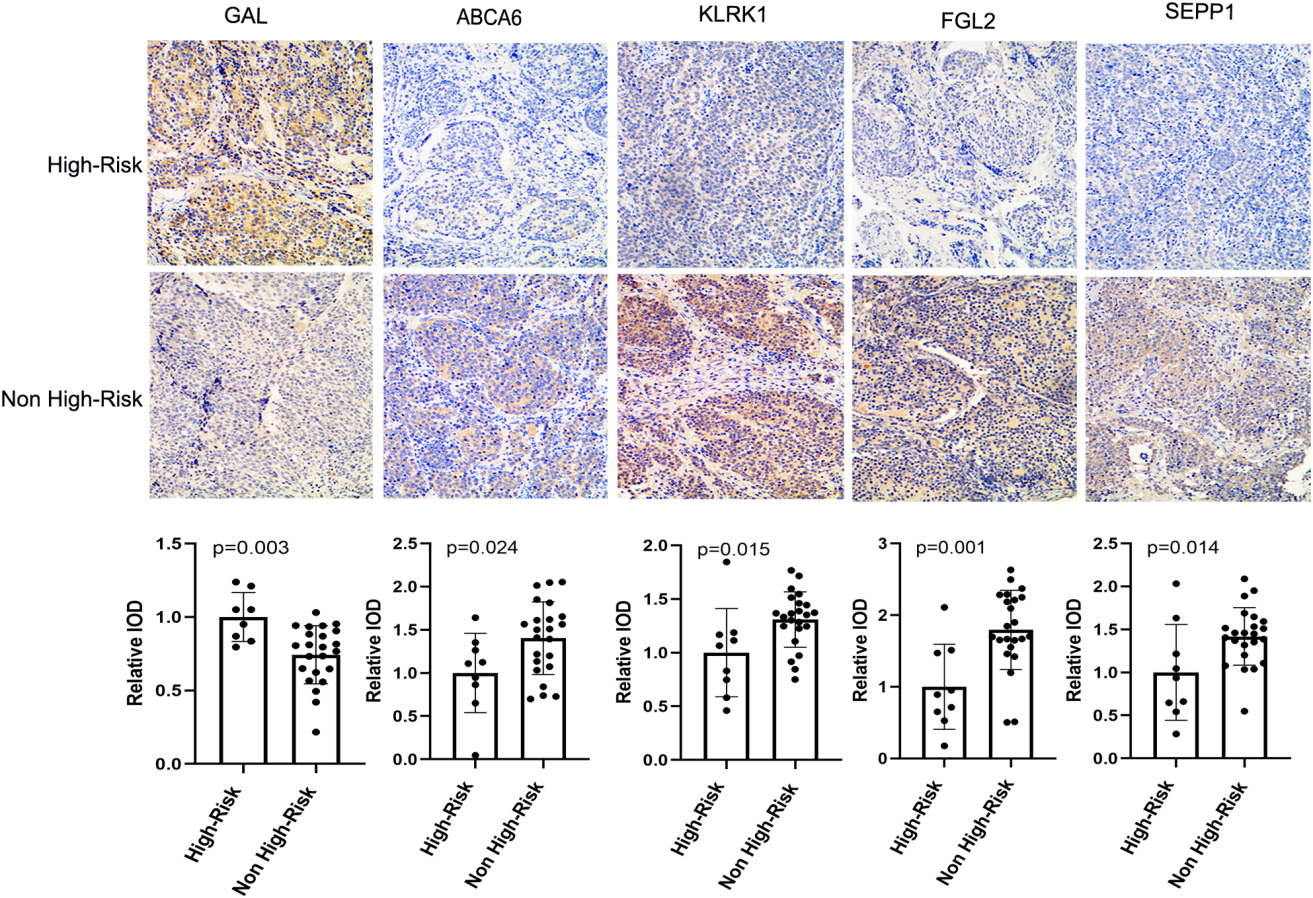
Immunohistochemical results and column diagram of relative IOD values of five prognostic model genes (GAL, SEPP1, FGL2, KLRK1, and ABCA6) in the High-Risk (n=7) NB and Non High-Risk (n=25) NB samples.

**Figure 8:**
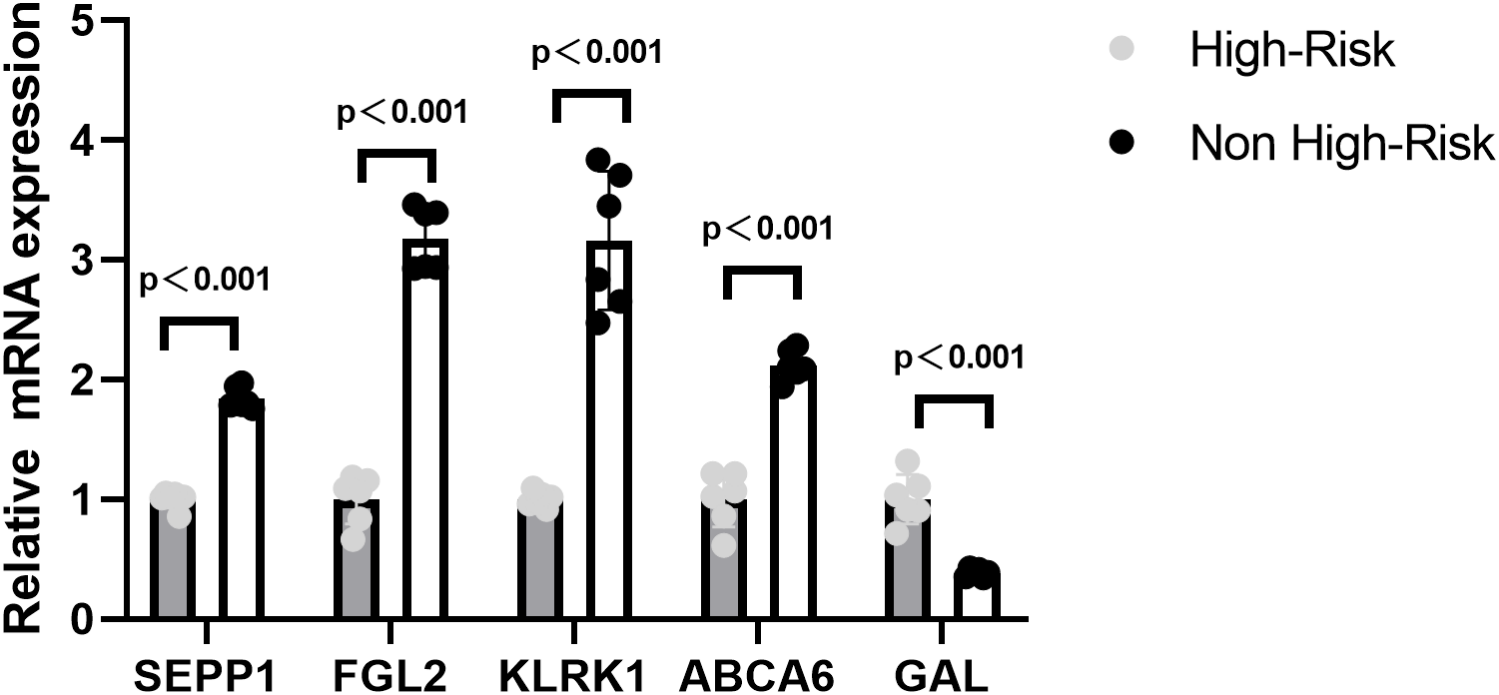
Relative mRNA expressing of five prognostic model genes (GAL, SEPP1, FGL2, KLRK1, and ABCA6) expression in the High-Risk NB and Non High-Risk NB samples by the quantitative real-time PCR.

In order to verify the expression of prognostic model genes, we measured the protein and mRNA levels of the five prognostic model genes. Immunohistochemistry staining showed that the level of SEPP1, FGL2, KLRK1, and ABCA6 were lower in the high risk NB samples, while the expression of GAL was higher in the high risk NB samples(p < 0.05, **Figure 7**). Real-time PCR analysis indicated the mRNA expression of SEPP1, FGL2, KLRK1, and ABCA6 was downregulated in high risk NB samples and GAL was upregulated in the high risk NB samples, which were all consistent with the results of our bioinformatics analysis (p < 0.05, **Figure 8**).

## 4 Discussion

NB is an embryonic childhood solid tumor arising from the peripheral sympathetic neural lineage that can occur in the paravertebral sympathetic ganglion of the chest, adrenal medulla, abdomen or pelvis (27). As the most common extracranial malignancy in children, NB accounts for approximately 7%-10% of all childhood cancers and 15% of the total cancer-related mortality. Although comprehensive treatments are applied to patients with NB, they still suffer from progression, metastasis, and poor prognosis (27). Statistically, the overall survival rate of patients with high-risk NB is less than 50% (28). In recent years, it has been reported that systemic inflammatory reactions, immune responses, and the TME may play critical roles in tumor progression, occurrence, and metastasis of NB (29–32). The TME comprises immune cells, extracellular matrix, signaling molecules, and surrounding blood vessels that can interact with cancer cells and influence cancer progression or metastasis (5, 33–35). Schaafsma et al. reported that immune-related B cells play an important role in the TME of NB that B cells can be used as prognostic biomarkers to predict progression and overall survival (36). Bao et al. verified that immunogenomic determinants of the TME were related to the prognosis of high-risk NB, and the crosstalk between cancer cells and components of the microenvironment could influence the phenotype of NB (37). In addition, reports have revealed that peripheral inflammatory immune cells in NB patients could influence anti-tumor immune effects and promote tumor cell migration and proliferation by changing the TME (4, 30, 38, 39).

In recent years, with the increase in research on different tumors, the role and mechanism of NETs in tumors have become clear (13, 40, 41). NETs can degrade the extracellular matrix and regulate the TME through the secretion of matrix metalloproteinases and proinflammatory cytokines, which may facilitate progression and metastasis. In contrast, NETs can induce epithelial-to-mesenchymal transition (EMT) in tumor cells, thereby facilitating tumor cell migration and invasiveness. Moreover, NETs can awaken dormant tumor cells and entrap circulating cancer cells, preventing tumor cells from being attacked by immune cells and promoting invasion and metastasis (12). Another important role of NETs in cancer is alteration of coagulation (42). NETs stimulate thrombosis in tumors and promote tumor metastasis (43). Richardson et al. found that patients with colorectal cancer had a significantly higher number of NETs than healthy volunteers. Patients with increased preoperative NETs production have a poorer prognosis (44). Xia et al. demonstrated that abdominal infectious complications after gastrectomy in gastric cancer (GC) patients would stimulate neutrophils to release more NETs, which could be dependent on the TGF-β signaling pathway to promote tumor cell proliferation, migration, and EMT (45). Moreover, NETs were found to be abundant in hepatic metastases in patients with colon and breast cancer. NETs could be predictive factors for the occurrence of hepatic metastases in patients with early stage breast cancer (46). However, the role of NETs in NB development and prognosis remains unclear. In our study, bioinformatics analysis was first performed to explore NET-related biomarkers and to construct a NETs prognostic risk model to predict the prognostic value and tumor-associated immune microenvironment in patients with NB.

In this study, we performed clustering analysis of NETRGs in NB for the first time. The consensus clustering analysis results showed that the NB sample was divided into two subtypes, with 125 DEGs between the subtypes. Five key genes (SEPP1, FGL2, KLRK1, ABCA6, and GAL) were screened for model construction and validation. Based on the expression of the above biomarkers, the samples in the GSE85047 and external validation set (TARGET) were classified into high- and low-risk groups using the risk score. The results showed that the survival rate of the low-risk group was higher, and it was proven that the risk score could better predict the survival status of patients with NB. Univariate and multivariate Cox analyses showed that the risk score could be an independent prognostic parameter of NB patients with NB, and the risk score with other clinical parameters could improve the value of the nomogram for prognosis. Finally, based on the above studies, we established and validated an NET-related stratification system that is beneficial for predicting the clinical outcome of NB, which is consistent with other tumors. Compared with the Koster’s original study in the GEO database from which our studies obtained data, our research methods were different from Koster’s study (47). Koster’s study used a different strategy to screen genes. They first collected genes with a higher coefficient of variation (CV > 0.1) in each dataset, which represented greater variability between samples (#TARGET = 3401; #NRC = 2853). They then selected genes that were common in both datasets (#common = 2435) for analysis. In our study, we focused on the expression of 136 genes closely related to Neutrophil Extracellular Traps (NETs). Using R package ‘ConsensusClusterPlus’ and ‘ pam ‘(Partitioning Around Medoids) clustering method for a consensus clustering analysis, the data is successfully divided into two unique subtypes. The two subtypes showed significant differences in the expression of NETs-related genes, reflecting different biological characteristics and underlying disease mechanisms. The results of the Koster study showed that 10 protein transcription modules centered on the positive feedback loop of TEAD4-MYCN became regulatory drivers of high-risk subtypes associated with the expansion of invasive neuroblastoma subtype (MYCN) (47). The five key genes we obtained were not directly related to the genes in the TEAD4-MYCN feedback loop, but might be indirectly related. Further research is needed to uncover the specific mechanism.

KLRK1 and Galectin among the five NETRGs in this study has and proven played a vital role in promoting immune escape by suppressing the immune microenvironment and affecting immune cell regulation and radiation resistance in NB (48). Therefore, we speculated that NETRGs might affect the release of NETs by regulating the immune microenvironment and function of immune cells, thus affecting the prognosis of NB. As a homodimeric lectin-like receptor, the NK cell lectin-like receptor K1 (KLRK1) gene encoding NKG2D is expressed in human NK cells. Studies have begun to emerge showing the protumor effects of tumor-associated neutrophils (TANs) in tumorigenesis, which may involve dysfunction of NK cells (49, 50). Rui Sun, et al. have proved that the neutrophils can decrease the responsiveness of NK-activating receptors(NKG2D)(50). So there might be a connection between the KLRK1 and tumor-associated neutrophils (TANs) (51–53). Studies have shown that KLRK1 activates NK cells and inhibits lung cancer proliferation and metastasis by controlling lung cancer through immune surveillance, thereby improving the prognosis, which is consistent with our findings (54). In our study, eight different immune cells, including activated NK cells, were associated with the immune microenvironment. Therefore, we hypothesized that KLRK1 might intervene in NET function by activating NK cells in NB.

GAL(Galectins)are a 15-member family composed by *β*-galactoside-binding proteins. By decorating the cell membrane and forming an extracellular molecular with galactoside units, GAL could play its biological role (55). There had reports that GAL levels in serum and tissue were associated with adverse clinical features of NB which was consistent with our bioinformatics analysis results (56). Galectin-1 could promote tumor cell proliferation by inducing angiogenesis and to induce the tumor cell immune escape (57, 58). Furthermore, there also had been reports that GAL existed in NETs while the neutrophils were undergoing NETosis. GAL immune complex could be observed in spontaneously NETotic cells of systemic lupus erythematosus (SLE) patients (59). Although there was no study about the association between NETs and NB, we speculated that GAL might affect the prognosis of NB patients by GAL gene regulation.

To date, there have been no experimental studies on NB retrieved from the other three NETRGs (SEPP1, FGL2, and ABCA6), but the three NETRGs have been proven to play a significant role in other types of tumors. Selenoprotein P1 (SEPP1), synthesized by the liver, is a secretory glycoprotein with antioxidant effects in some diseases, including inflammatory bowel disease cancer and so on (60). Studies have demonstrated that SEPP1 silencing increases the release of inflammatory cytokines and inhibits adipocyte differentiation, leading to significant oxidative stress and inflammatory response (61). SEPP1 has also been reported to control the production of free radicals and reduce oxidative damage, thereby inhibiting prostate cancer (61). SEPP1, a cancer suppressor gene in other cancers, is consistent with our biological results. The role of increased oxidative damage in the development of malignancy is well characterized, and reactive oxygen species (ROS) are a well-known contributor to a chronic inflammatory microenvironment (62). When Some kind of stimulus occurs, the neutrophils can produce huge amounts of reactive oxidative species (ROS). More and more studies were focused on the antioxidant capacity of SEPP1 in occurrences and development of malignant tumors by the ROS. Mithunan Ravindran,et al. have reporter that internal factors(e.g. reactive oxygen species (ROS) production and transcription factor activation) have all been demonstrated to influence specific Neutrophil extracellular traps (NETs) pathways s (63). Therefore, we can infer that there might be a connection between the SEPP1 and tumor-associated neutrophils (TANs) or NETs. We hypothesized that oxidative stress and inflammatory response might be the bridge between SEPP1 and NETS in NB.

The ABCA subfamily of transporters is composed of 12 members that regulate cellular lipid transport and maintain lipid homeostasis. The expression of ABCA6 can impair intracellular levels of cholesterol, which is important for regulating the membrane fluidity of cancer cells (64). Pasello et al. revealed that ABCA6 functions as a tumor suppressor via cholesterol-mediated inhibition of IGF1R/AKT/MDM2 signaling in EWS (65). The expression of ABCA6 in NB and its association with NETs remains unclear and requires further study.

Fibrinogen-like protein 2 (FGL2), a member of the fibrinogen superfamily, is mainly expressed in macrophages, neutrophils, regulatory T cells, endothelial cells, and tumor cells (66). Moreover, FGL2 is a pleiotropic immune regulator of both the innate and adaptive responses. Although no reports have been published on the use of FGL2 in NB, there have been some studies on the association between FGL2 and NETs or other tumors. Li et al. showed that neutrophil-FGL2 could promote NETs formation in fulminant viral hepatitis, and that increased plasma NETs were associated with coagulation dysfunction in patients with acute liver injury (67). Olli-Pekka Pulkka et al. proved that high expression of FGL2 in gastrointestinal stromal tumors was associated with favorable survival outcomes with better recurrence-free survival, small size, and low tumor-infiltrating lymphocytes (68). Yuan et al. proposed that FGL2 enhances T cell-mediated anti-tumor responses in lung adenocarcinoma (69). Previous studies have shown that FGL2 is positively correlated with CD8^+^ T cells, which could play an important role in the antitumor immune response by changing the TME. Feng et al. analyzed several online databases and demonstrated that low FGL2 expression in patients with breast cancer was associated with adverse prognosis, and high FGL2 was positively associated with antitumor immune cell infiltration (70). Hence,we hypothesized that FGL2 could control the infiltration of anti-tumor immune cells, which might influence the formation and function of NETs and play a role in the prognosis of NB. However, it has been reported that FGL2 in different cancer models may have inconsistent roles. In contrast, FGL2 may play a role in accelerating cancer progression by activating cancer-associated fibroblasts in the TME or by inducing epithelial macrophage transformation. Patients with FGL2 overexpression have an adverse prognosis in human glioma, clear cell carcinoma, and other cancers (71). We assumed that these different results in various types of cancer might be due to the different functions of the mFGL2 and sFGL2 subtypes, which should be assessed when studying the role of FGL2 in other cancers.

We performed GO and KEGG enrichment analyses based on the DEGs between the two subtypes. GO analysis suggested that DEGs were involved in many immune-related biological processes and functions, especially neutrophil-related items such as the regulation of neutrophil migration and neutrophil differentiation. Therefore, our study concluded that NETs are closely associated with the TME and immune infiltration. KEGG pathways were enriched in several classical immune-related signaling pathways and tumor signaling pathways, including ‘Complement and coagulation cascades,’ ‘Primary immunodeficiency’ ‘Neutrophil extracellular trap formation’, ‘T cell receptor signaling pathway,’ ‘PD-L1 expression and PD-1 checkpoint pathway in cancer’ and so on. We speculated that immune infiltration may be closely related to tumor prognosis. In summary, NETs play a vital role in tumor immunity, which is closely associated with the prognosis of patients with NB.

The TME plays a vital role in the occurrence and metastasis of tumors. Our analysis showed that the three scores, including the ESTIMATE, immune, and stromal scores, were significantly higher in the low-risk group, which is in accordance with other studies. In addition, 8 differential immune cells between the two risk subgroups. Plasma cells are specialized antibody-secreting, terminally differentiated CD38^+^ B cells that are components of tumor inflammatory infiltrates (72). In our study, plasma cells had the strongest positive association with risk score, which might indicate a high level of antitumor immune response in the high-risk groups. In contrast, there was a strong negative relationship between the risk score and CD4 memory resting T cells. As the main force in the antitumor immunity of NB, T lymphocytes (CD8^+^ T cells or resting memory CD4^+^ T cells) can significantly inhibit tumor development and improve prognosis, as expected (73). These findings might indicate a lower level of antitumor immune response in the high-risk groups. Finally, we speculated that NETRGs could influence immune cell infiltration, which could change the TME and determine the prognosis of NB patients.

However, our study has some limitations. All the NB information in our study was obtained from a public database of retrospective data. Our research mainly focuses on analysis at the gene expression level, but the formation of NETs is a complex biological process involving the regulation of multiple cytokines and cellular interactions. Therefore, future studies should be combined with more direct biological evidence, such as detection of NETs specific markers (MPO/citH3, etc.) by immunohistochemistry or flow cytometry, to more accurately assess the role and explore the mechanism of NETs in NB. Additional validation research should be conducted using *in vivo* and *in vitro* experiments and larger multicenter large samples.

## 5 Conclusion

In conclusion, we initially found that five genes, SEPP1, FGL2, KLRK1, ABCA6, and GAL, were significantly differentially expressed in NETs-associated molecular isoforms, and two Netrg molecular isoforms were found to be associated with poorer prognosis. This stratification might provide insight into the prediction of prognosis and ideal immunotherapy strategies for patients with NB. However, we also noted that these genes are not only associated with NETs but may also be involved in other immune cell activation processes. Therefore, the exact roles of these genes and their specific mechanisms in the formation of NETs and the development of NB still need to be further investigated.

## Data Availability

The original contributions presented in the study are included in the article/**Supplementary Material**. Further inquiries can be directed to the corresponding author.

## Supplementary material

Supplementary Figure 1 K-M curves of five prognostic model genes in the High-Risk (n=134)and low (n =135) Risk.
