# Supplementary figures and images for: Exploring the role of neutrophil extracellular traps in neuroblastoma: identification of molecular subtypes and prognostic implications
Source: Front Oncol. 2024 Nov 7;14:1361871. doi: 10.3389/fonc.2024.1361871 (PMC11578966; doi:10.3389/fonc.2024.1361871)

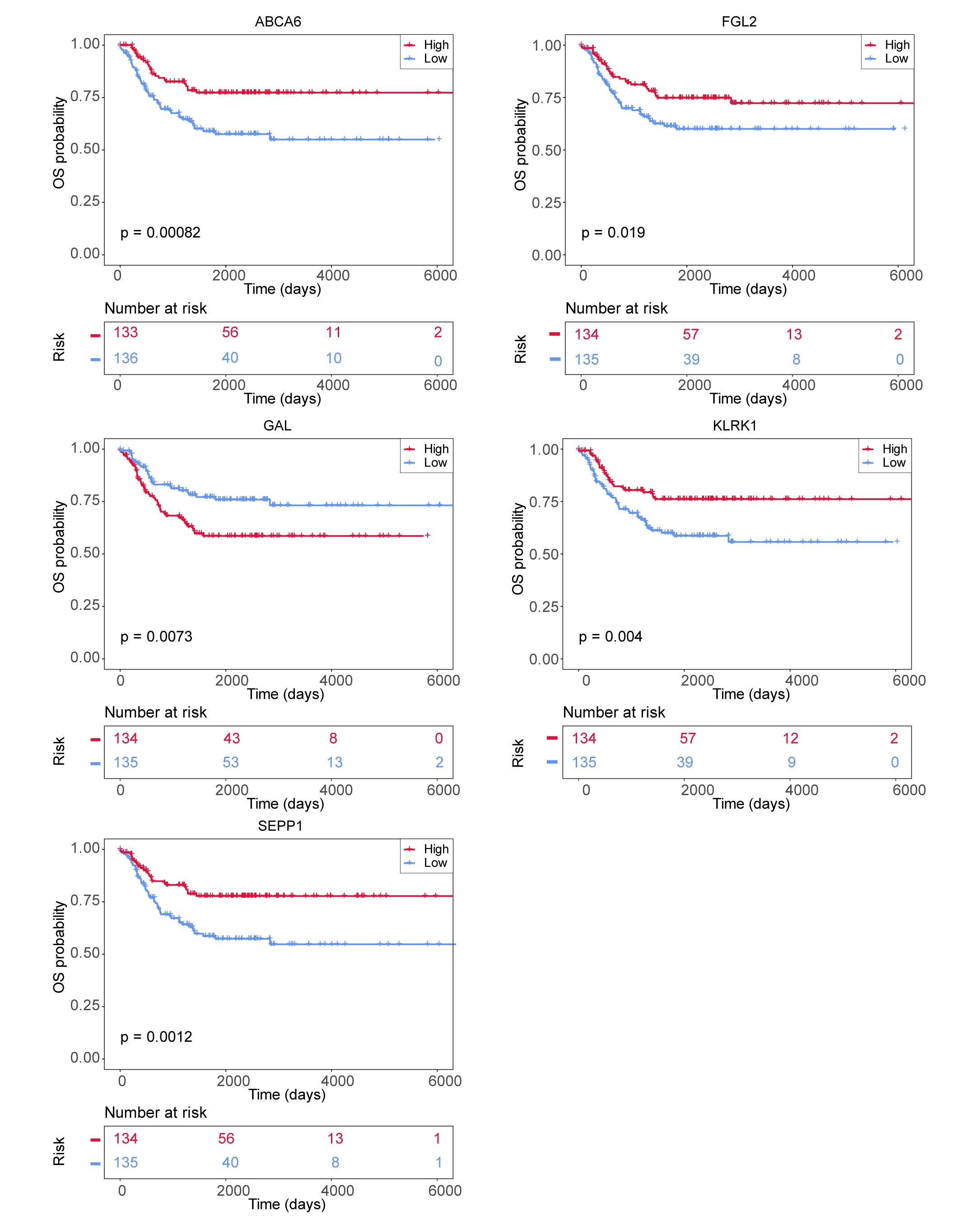

Supplement: Supplementary Figure 1 — K-M curves of five prognostic model genes in the High-Risk (n=134)and low (n =135) Risk. [file Image1.tif]
