# Supplementary material for: Exploring the role of neutrophil extracellular traps in neuroblastoma: identification of molecular subtypes and prognostic implications
Source: Front Oncol. 2024 Nov 7;14:1361871. doi: 10.3389/fonc.2024.1361871 (PMC11578966; doi:10.3389/fonc.2024.1361871)
Supplement: Supplementary file 5 [file DataSheet1.docx]

logFC AveExpr t P.Value adj.P.Val B change

C1S 1.67658112078968 7.24235775144928 18.8060021350239 2.04325623996266e-51 1.75699604074389e-47 106.299430621108 up

CYBB 1.37972346996052 8.68778056123188 18.5268528726104 2.06828735221185e-50 7.49979919518762e-47 104.00490064649 up

CD53 1.54192106775467 8.7088223115942 18.4985256256907 2.61651326730583e-50 7.49979919518762e-47 103.771824539973 up

C1orf162 1.152763404617 7.83672560036232 18.0483928691599 1.10284977403967e-48 2.37085130174178e-45 100.062934499114 up

LAIR1 1.00292771036589 6.63676731304348 17.838787510318 6.31478225921688e-48 1.08601625294012e-44 98.3328935853224 up

HLA.DMA 1.24055760471703 8.32864057101449 17.7147110679798 1.77536092366295e-47 2.31950597868905e-44 97.30802266932 up

CTSS 1.3182470964517 6.64173914528986 17.7073172672304 1.88818953957708e-47 2.31950597868905e-44 97.246933193681 up

C3 2.18521886575941 7.41646183369565 17.5036117368155 1.03171717023831e-46 1.1089669933599e-43 95.5631862771387 up

HLA.DRA 1.29231218344828 10.1844501253623 17.4346778868302 1.83336918323381e-46 1.75168240073639e-43 94.9931323131048 up

CD74 1.39098686138984 9.97998152572464 17.2647980567632 7.56477124443747e-46 6.50494679309178e-43 93.5877815731981 up

C1R 1.56268362135825 7.26344420543478 17.2305684994346 1.00660059372171e-45 7.86887136855722e-43 93.3045331132965 up

CTSH 1.31518927586733 6.76762310905797 17.1972854789139 1.32891637247309e-45 9.18523015860678e-43 93.0290937247977 up

DOCK2 1.23810703413003 6.24143895036232 17.1920196571946 1.38862649217221e-45 9.18523015860678e-43 92.9855134518765 up

IFITM1 1.38181412535404 8.42767521050725 17.1574257004876 1.85348379351483e-45 1.13843622431672e-42 92.699198179932 up

CSF1R 1.10055894783364 6.74249260543478 17.1254287900049 2.42095177231429e-45 1.38785095267537e-42 92.4343570746106 up

FGL2 1.27683124550145 7.45501935978261 17.1127837730396 2.69048029495987e-45 1.44596500352249e-42 92.3296880736012 up

LAPTM5 1.24554985318768 9.57595150108696 17.0724236164053 3.76843099151736e-45 1.90538544557076e-42 91.9955886503238 up

AOAH 1.4717192496815 6.81662494456522 17.0656259662524 3.98847982559295e-45 1.90538544557076e-42 91.9393153281989 up

LCP2 1.19733111993683 7.37270399710145 17.055572613787 4.33769771390967e-45 1.96315066536364e-42 91.8560887816973 up

CTSC 1.06232160067912 8.26573229673913 16.8777887126554 1.9141446335447e-44 7.48169531993222e-42 90.3840648716231 up

EVI2B 1.70066643212424 8.07340088478261 16.8448028338942 2.52122850469992e-44 9.42610604865851e-42 90.1109036216818 up

CD4 1.05185536749144 7.01821219021739 16.7603150553425 5.10578343262992e-44 1.75618526948739e-41 89.411199544443 up

NOTCH2 1.03830573428271 6.58409229601449 16.4460281389276 7.04944671474267e-43 2.33146893461816e-40 86.8080313753969 up

IRF8 1.29001474788629 6.9271607576087 16.2237016520716 4.51455882746864e-42 1.38645326276439e-39 84.9666122083904 up

FYB 1.59917886037905 6.85821325326087 16.0007592879322 2.9047309084982e-41 8.0573487361858e-39 83.1205419416337 up

HLA.E 1.01874725518821 8.14103208442029 15.9916696074735 3.13373790664088e-41 8.16576129066817e-39 83.0452898067464 up

HLA.B 1.05810255832588 10.9409538166667 15.9236950246616 5.5271393008086e-41 1.3124283013022e-38 82.4825837389096 up

GPR65 1.08508941590419 6.07366686268116 15.9197094679546 5.7141107359263e-41 1.3124283013022e-38 82.4495931954791 up

HLA.DMB 1.13993776607528 7.40138181376812 15.8715224841367 8.54324531098061e-41 1.79178942510054e-38 82.0507489980978 up

ARHGDIB 1.2175407978626 8.76530917898551 15.7274483874403 2.84281116743542e-40 5.55575755199481e-38 80.8585500889674 up

FPR3 1.1353871729034 6.81147022572464 15.6883665500022 3.93860434653773e-40 7.36262147301694e-38 80.535239949278 up

C3AR1 1.12527492245854 6.48701142427536 15.6484325002045 5.49547947599859e-40 1.00543889391727e-37 80.2049227904939 up

AHR 1.2353517305712 7.65998576884058 15.6283160200619 6.49936844723559e-40 1.16433477662039e-37 80.0385451688813 up

ALOX5AP 1.30392403771519 6.75917547971015 15.435529962291 3.24263028977835e-39 5.4673289925106e-37 78.4447136651521 up

GIMAP4 1.1515857762727 6.22389104094203 15.4126330678221 3.92431800481516e-39 6.36702085347275e-37 78.2555017181907 up

CYBRD1 1.38522498180048 8.05781371847826 15.3659387130854 5.79069342219829e-39 9.22114309953391e-37 77.8696969860919 up

CASP1 1.02311318849697 4.69583941485507 15.1410233282084 3.76752558874587e-38 5.78517009600459e-36 76.0126315575133 up

DAB2 1.33781704469071 7.90335090905797 15.1347070439075 3.97084253147442e-38 5.9903991102015e-36 75.9605124795351 up

DOCK8 1.19836040262701 6.03269986557971 15.0356902615501 9.04918890391879e-38 1.34162026525513e-35 75.1437277887292 up

EMP3 1.05922465304027 6.9708040634058 15.0217850540406 1.0158489619148e-37 1.48055681754328e-35 75.0290642199263 up

CD180 1.27374650426428 5.64262373007246 15.0007727126089 1.20978497562856e-37 1.67789371055322e-35 74.8558138536679 up

FCER1G 1.22400082636483 7.37952255362319 14.9296838421974 2.18446968500621e-37 2.8898853571336e-35 74.2698503634672 up

CXCL12 1.17868125776783 6.87999685615942 14.9255324509727 2.26114935657549e-37 2.94600353290797e-35 74.2356403539964 up

HLA.DPA1 1.3749709840695 10.1000340666667 14.9107666482247 2.55634046863226e-37 3.28089129698042e-35 74.1139689617658 up

GLIPR1 1.40851030196368 8.16971681086956 14.7771945401933 7.75226499546327e-37 9.52310381371266e-35 73.0139034033321 up

C17orf87 1.10772815587786 6.30002129673913 14.4328350104526 1.34669112872857e-35 1.46584772353633e-33 70.1832194816051 up

C7 1.98266013484601 7.49708120072464 14.3502944154954 2.66622015746682e-35 2.83047248568607e-33 69.5060142451368 up

ARHGEF6 1.04962503079758 5.88053047826087 14.3207525232843 3.40411925629684e-35 3.56736242295155e-33 69.2637696374813 up

CFH 1.55142231420374 6.20465109166667 14.3193678835796 3.44331993377112e-35 3.56736242295155e-33 69.2524172860039 up

CNN2 1.08457174036852 7.32498762065217 14.270043667423 5.17689680440425e-35 5.29953995488954e-33 68.8481224891741 up

GMFG 1.19554857231377 7.29463296413044 14.2451330390157 6.36025706481136e-35 6.35951750003638e-33 68.6440155601232 up

IFI16 1.26812628615425 7.48930155253623 14.2367021779721 6.81911824448097e-35 6.73995376830941e-33 68.5749486823502 up

GIMAP2 1.21956937709397 5.48980259746377 14.2196978088555 7.84764339792773e-35 7.66839608849779e-33 68.4356649002924 up

DCN 1.82635455133983 6.92612170181159 14.1811600508478 1.07887601301375e-34 1.04238818380957e-32 68.1200927504643 up

CCND2 1.17376902920242 6.8700129807971 14.1254836560215 1.70836090585746e-34 1.61430719005146e-32 67.664410357823 up

AMICA1 1.21368395924717 5.17496116268116 13.9816100348967 5.59528376016301e-34 5.11849415464273e-32 66.4882016008996 up

CD14 1.00412695797842 7.89407245942029 13.9250215859263 8.91752899816642e-34 8.07177177423506e-32 66.0261193962285 up

CCL2 1.35480890840221 8.73880386268116 13.7729800221354 3.11474991349593e-33 2.76120974290221e-31 64.7862104547603 up

HLA.DPB1 1.40392460133193 9.86101809166667 13.7699234586555 3.19398097968491e-33 2.80255535146026e-31 64.7613088564311 up

IL2RG 1.72791804803896 6.89204507065217 13.7452426777976 3.91205022109096e-33 3.36397198511611e-31 64.5602732576443 up

CD37 1.56320839660963 7.53829122318841 13.7115002116023 5.16139184076038e-33 4.39433746917807e-31 64.2855335966407 up

ARHGAP15 1.23351493131877 6.38881721884058 13.5987436453637 1.30192427467988e-32 1.07646604211272e-30 63.3683630890625 up

CYSLTR1 1.1015883719821 5.69633886884058 13.5041504412186 2.82613898276965e-32 2.31447324884155e-30 62.6000681784148 up

IL13RA1 1.0000967579363 6.56782437318841 13.369654728074 8.4917188974695e-32 6.51966882136967e-30 61.509549507448 up

IGJ 2.33659360227428 6.45644286666667 13.3311095389211 1.16345614456768e-31 8.69961685838039e-30 61.1974367288399 up

CCR1 1.18853337827849 5.45992126231884 13.2553385171159 2.15942172151343e-31 1.56040902380622e-29 60.5844575883201 up

ETS1 1.16212041196631 6.9874464576087 13.227001130914 2.72083548567379e-31 1.91774297879581e-29 60.3554067247801 up

ABI3BP 1.24030230639642 6.38973106521739 13.2211370386984 2.85407815103154e-31 1.99530227810733e-29 60.3080207926599 up

EVI2A 1.0951610775941 4.24886019528986 13.1955323575712 3.51644335260389e-31 2.43854003137426e-29 60.1011717779614 up

IL1R1 1.20477682266386 5.77166436050725 13.1882627851218 3.73104230273414e-31 2.56665862089687e-29 60.0424603483456 up

ABCA6 1.06070939593577 5.11164884710145 13.1537524639131 4.9421705266371e-31 3.36239968348952e-29 59.7638427615489 up

GIMAP7 1.15939712911819 6.28986198405797 13.0876724119314 8.46237841035566e-31 5.59753784235756e-29 59.2308109451896 up

DENND2D 1.42387686403264 6.92819182355072 12.9787402293559 2.05095582982847e-30 1.3063829022737e-28 58.3534765918173 up

SEPP1 1.19602184140037 8.13407069347826 12.9592561171069 2.40239907333598e-30 1.51898747291295e-28 58.1967356631189 up

CD48 1.36774870987102 7.37890071014493 12.9026706208304 3.80183184429431e-30 2.35193899489833e-28 57.7418530056536 up

IGF1 1.24466963387207 7.67741084565217 12.8673117011849 5.06355246841166e-30 3.11010626256228e-28 57.4578544090939 up

BIRC3 1.3518920585154 4.8593317192029 12.8626596089051 5.25804690246989e-30 3.20666278825096e-28 57.4205036490708 up

GIMAP5 1.0264977286707 6.91678083007246 12.8398108780367 6.327041697337e-30 3.81987479534228e-28 57.2371041450456 up

A2M 1.05806716744933 8.65421386485507 12.7610447550867 1.19677725935503e-29 7.0007399001319e-28 56.6055002468467 up

FBLN5 1.51572475168202 6.88882389927536 12.7135557749502 1.75672194787489e-29 1.00040079667392e-27 56.2251769301312 up

LCP1 1.21380199137668 8.52930021811594 12.635771097904 3.29149947597658e-29 1.81433358935402e-27 55.6030205325239 up

EFEMP1 1.64339984273756 7.34531146666667 12.4321795763299 1.69456782264057e-28 8.52139690461183e-27 53.9794566589359 up

CX3CR1 1.33030423407739 5.40009667137681 12.4220202786725 1.83861315078351e-28 9.1919967927834e-27 53.8986301319896 up

GPNMB 1.14283027711503 9.05718965144928 12.3538275100028 3.17770078211607e-28 1.56143137288092e-26 53.356576065598 up

CTSK 1.13968739497763 7.68727062173913 12.259458846505 6.76623000591445e-28 3.23237843449213e-26 52.6078633430679 up

ALDH1A1 1.10730422510134 6.01814742681159 12.2470015845741 7.47519653117113e-28 3.55133784373152e-26 52.5091530854093 up

GAPT 1.24253730508028 5.0135959057971 11.9288128808876 9.43207918285104e-27 4.05532244466681e-25 49.9981484949621 up

CD96 1.34322871807844 6.06541095253623 11.8886352556816 1.29719553234086e-26 5.5220714765342e-25 49.6825474846356 up

LEF1 1.40482988676494 6.76143715253623 11.8434808301956 1.85512546098843e-26 7.74379798011628e-25 49.3282604820548 up

GBP1 1.00610732858647 6.23863094021739 11.8261719125301 2.12755512816415e-26 8.75351509429832e-25 49.1925681454639 up

CD2 1.0589790612319 5.51666349673913 11.8059028183048 2.49764340137207e-26 1.02272550516183e-24 49.0337516842579 up

IFI30 1.06432512518558 8.7573113692029 11.7732346818354 3.23374954529689e-26 1.31165152547207e-24 48.7779712678955 up

GZMK 1.34785169043959 5.32561147427536 11.6735657265952 7.10120577298446e-26 2.77560311099515e-24 47.9990484535654 up

CD163 1.00849493700448 5.72869627681159 11.5150849330357 2.46928705201162e-25 9.15232731045168e-24 46.7651331247993 up

GPR174 1.95769689807318 6.16767115905797 11.5081407652752 2.60753667882667e-25 9.62326519366116e-24 46.711199748074 up

ANXA1 1.1313569834588 9.24385677101449 11.490217152763 3.00105515036164e-25 1.09813077608339e-23 46.5720447138593 up

ISLR 1.25112245698342 7.10937974782609 11.2640755063753 1.75640300111968e-24 6.11470016462679e-23 44.8229910671978 up

CD3E 1.54040647142932 6.08391911884058 11.0840888318576 7.10331635460922e-24 2.38599286458143e-22 43.4400798764893 up

IFI44L 1.59272931242432 7.1371144076087 10.905368544749 2.82117929424392e-23 9.12004539518927e-22 42.0753584191311 up

CCDC80 1.42793815418268 6.86037628152174 10.814861888955 5.65376105054337e-23 1.76787968267718e-21 41.3875836441356 up

KLRK1 1.10046372257963 5.30920064637681 10.7096930615654 1.26449154507773e-22 3.8972626509403e-21 40.5912996933086 up

CD52 1.34318093981574 8.01295090507246 10.6602097117184 1.84470878841257e-22 5.62505350055308e-21 40.2177419522267 up

COL14A1 1.02643052754409 5.82387215108696 10.6308211680934 2.30776406121897e-22 7.00609533567337e-21 39.9962227411235 up

CD3G 1.63619896293761 6.11115680797101 10.550546496706 4.24923972506174e-22 1.23443285120966e-20 39.3924505439457 up

CD69 1.22925690795999 4.96402297717391 10.5078110996599 5.87646367348584e-22 1.69002378355534e-20 39.0718133539012 up

FCGR2A 1.15178740136352 8.06391303586956 10.4968306156118 6.3864065260424e-22 1.83055699058129e-20 38.989518014854 up

F13A1 1.48698459435115 6.42001814021739 10.458232554258 8.55404496834278e-22 2.42759843837556e-20 38.7005301724814 up

IL7R 1.80734384422743 6.28771711630435 10.437595161759 9.99884459826903e-22 2.80065357330669e-20 38.54620382285 up

ITK 1.1778655098289 5.66327846086956 10.2394873920545 4.4431958472256e-21 1.18288052911124e-19 37.071555949694 up

GPR171 1.01366019036062 4.23739253224638 10.0137483264876 2.39434292768646e-20 6.00261073911833e-19 35.4067704634726 up

IFIT3 1.02829994110029 6.02367630869565 9.93244474732707 4.37377517402741e-20 1.0624319977814e-18 34.8113829357019 up

CD3D 1.37064424750197 6.10190829818841 9.74098142604302 1.79138361336863e-19 4.14088916434323e-18 33.4184347834115 up

CCL21 1.80231996196368 7.71574656702899 9.72441001530047 2.02267125684374e-19 4.63812003669316e-18 33.2984894093072 up

APOD 1.61711826428008 6.82136997826087 9.54266450282525 7.61203005179323e-19 1.6655431657855e-17 31.9896227164577 up

CCL19 1.69699175447223 7.71319208043478 9.53518271865924 8.03679338291264e-19 1.74957939999154e-17 31.9360055368781 up

CXCL10 1.4966883317294 6.07335987391304 8.95862775198028 4.94652343198201e-17 9.1670592654339e-16 27.87039760857 up

FAIM3 1.22534486535404 6.53548215724638 8.89010477226331 8.00019685615327e-17 1.4605879568166e-15 27.3962806283637 up

CXCL13 1.03139805770992 3.85402537753623 8.40200995576806 2.31742016149719e-15 3.70399553321828e-14 24.0791020632406 up

CCR7 1.28838980849697 6.83687075507246 8.32163358893266 3.99301656444903e-15 6.30017420875178e-14 23.5433453017383 up

CCL18 1.20546621607265 7.49842438152174 6.97255752338357 2.28104865260123e-11 2.58428687269012e-10 15.046543515441 up

CXCL9 1.19106331295604 5.53406758550725 6.8558095629989 4.60358984122853e-11 5.12112148055939e-10 14.3590476815116 up

LOC100510059 1.55265241155041 7.20687276594203 6.84873882380958 4.80236965522237e-11 5.33534582238465e-10 14.3176734968762 up

CXCL11 1.15020001257173 5.98828538695652 6.7017529877208 1.14867614920721e-10 1.23777772017955e-09 13.4644936225793 up

KCNH5 -1.05213119783627 5.18170989891304 -6.02048834805036 5.50205009697688e-09 5.08186130869003e-08 9.68898049021002 down

GAL -1.15664605426165 7.43399304456522 -5.44377290095589 1.14949653263577e-07 9.18635751313664e-07 6.73766474664985 down
